# Inkjet Printing of Drug-Loaded Mesoporous Silica Nanoparticles—A Platform for Drug Development

**DOI:** 10.3390/molecules22112020

**Published:** 2017-11-21

**Authors:** Henrika Wickström, Ellen Hilgert, Johan O. Nyman, Diti Desai, Didem Şen Karaman, Thomas de Beer, Niklas Sandler, Jessica M. Rosenholm

**Affiliations:** 1 Pharmaceutical Sciences Laboratory, Åbo Akademi University, FI-20520 Turku, Finland; henrika.wickstrom@abo.fi (H.W.); ellen@hilgert.be (E.H.); johan.o.nyman@abo.fi (J.O.N.); diti.desai@gmail.com (D.D.); didem.sen@abo.fi (D.S.K.); niklas.sandler@abo.fi (N.S.); 2 Laboratory of Pharmaceutical Process Analytical Technology, Ghent University, B-9000 Ghent, Belgium; Thomas.DeBeer@ugent.be

**Keywords:** mesoporous silica nanoparticles, digital printing, inkjet printing technology, pharmaceutical nanosuspension, drug delivery, print morphology, screening platform

## Abstract

Mesoporous silica nanoparticles (MSNs) have shown great potential in improving drug delivery of poorly water soluble (BCS class II, IV) and poorly permeable (BCS class III, IV) drugs, as well as facilitating successful delivery of unstable compounds. The nanoparticle technology would allow improved treatment by reducing adverse reactions of currently approved drugs and possibly reintroducing previously discarded compounds from the drug development pipeline. This study aims to highlight important aspects in mesoporous silica nanoparticle (MSN) ink formulation development for digital inkjet printing technology and to advice on choosing a method (2D/3D) for nanoparticle print deposit characterization. The results show that both unfunctionalized and polyethyeleneimine (PEI) surface functionalized MSNs, as well as drug-free and drug-loaded MSN–PEI suspensions, can be successfully inkjet-printed. Furthermore, the model BCS class IV drug remained incorporated in the MSNs and the suspension remained physically stable during the processing time and steps. This proof-of-concept study suggests that inkjet printing technology would be a flexible deposition method of pharmaceutical MSN suspensions to generate patterns according to predefined designs. The concept could be utilized as a versatile drug screening platform in the future due to the possibility of accurately depositing controlled volumes of MSN suspensions on various materials.

## 1. Introduction

Printing technologies are nowadays used for the design and manufacture of products by deposition of different materials with electrical, optical, chemical, biological, and structural functionalities [1,2]. In particular, digital (2D and 3D) printing technologies are appealing for pharmaceutical applications since state-of-the-art technologies enable the deposition of different liquid, semi-solid, and solid materials containing one or several active pharmaceutical ingredients (APIs) according to predefined designs [3,4,5,6]. Consequently, printing technologies can be used to manufacture personalized doses and drug delivery systems. The possibility of late-stage customization of the medicine according to a patient’s specific needs as well as the flexibility in production volumes make the manufacturing method applicable for decentralized manufacturing of pharmaceuticals at the point of care [7].

The drug discovery approach, utilizing combinational chemistry and high-throughput screening, has resulted in vast libraries of poorly water-soluble and poorly permeable lead compounds and drug candidates [8,9]. Consequently, different formulation strategies have been developed to overcome the hurdles of poor drug delivery and poor bioavailability [10]. Formulation approaches utilizing nanoparticles in combination with printing technologies have been conducted to address this issue. Pharmaceutical nanosuspensions have been developed and deposited by means of roll-to-roll [11,12] and drop-on-demand (DoD) printing technologies [5,13,14,15]. Accurate deposition of antibacterial agents onto implant materials, development of transdermal/mucosal and oral, immediate and controlled release drug delivery systems have been demonstrated to be doable using inkjet technology. Furthermore, the possibility of printing different geometries has been shown to be of importance for tailoring the drug release from the printed drug delivery system [16,17]. The main reason for the development of the polymeric nanoparticles and cyclodextrin inclusion complexes has been to improve and control the delivery of especially potent and poorly soluble drugs. Apart from the manufacture of pharmaceuticals and modification of implants, printing of nanosuspensions has been used for labeling, diagnostic, and screening applications; Quick Response (QR) codes have been printed using RGB (red, green, blue) nanoparticle inks for anti-counterfeit purposes on capsules [18] and nanoparticle-based lab-on-a-chip platforms have been developed by combining microfluidics, electronics, and inkjet printing [19]. Inkjet printing has also been utilized as a screening platform in combination with a separate detection method for rapid, low-cost, high-throughput screening of chemical libraries and novel biomaterials [20,21]. Systematic investigations concerning the development of inkjet printable nanosuspensions have been conducted due to interest in inkjet-printed electronics [22,23]. The composition of the nanosuspensions with regard to the physicochemical properties of the particles (size, polydispersity and net surface charge), particle concentration, excipient addition (surfactants), and solvent system, has an impact on the performance of the ink formulation [2,23,24]. Furthermore, the interplay between the ink formulation and the substrate must also be considered since it dictates the final deposit morphology of the printed product [25,26,27,28]. Previously, investigations regarding inkjet-printed drop morphologies of monodisperse silica and silicon dioxide microspheres onto various hydrophilic and hydrophobic materials have been performed [25,26,29].

Utilization of nanomaterials has proven to be favorable in the fields of drug delivery, in vitro diagnostics, and in vivo imaging, and for the development of biomaterials, implants, and coatings [30,31]. These materials have shown to enable improvement or added functionality of previous compounds and could also have potential in the development of super generics [32]. Such studies showing improved delivery and usability of poorly soluble, poorly permeable, very potent APIs [33], biomolecules [34], proteins [35], peptides [36], antibodies [37], and genes [38] have been published to date.

The FDA-approved hydrophobic colloidal silica material has already been widely used in different pharmaceutical formulations. However, the first proof-of-concept study using ordered mesoporous silica as drug delivery vehicle of a poorly soluble drug was only conducted recently in human [39]. Mesoporous silica nanoparticles (MSNs) have still not been studied in man, but have been introduced as a versatile and biocompatible drug delivery platform [40,41,42]. Immediate and controlled release MSN-based drug delivery systems have been developed for oral [33,43,44,45], transdermal [46], and intravenous (i.v.) [38] administration of compounds with poor stability or poor solubility. Great potential has been seen in the application of MSNs as targeted drug delivery vehicles for anti-cancer drugs [37]. Furthermore, uni- and dual-stimuli-responsive MSN drug delivery mechanisms have proven to be possible, having, i.e., a change in pH as a trigger for the cargo deposition [47]. The versatility in drug delivery described above is enabled by the design and control of key features of the mesoporous material such as particle size, particle surface, and pore size, shape, and volume. Tailoring the surface properties of the particles allows for extensive design opportunities. An essential property of the functionalization is the improved dispersibility of the nanoparticles in media and the benefit seen in studies investigating the cellular uptake of the functionalized particles [38,48].

Biopharmaceutics Classification System (BCS) class IV drugs have poor water solubility and poor permeability. Thus, formulation strategies including MSNs could possibly address these issues by facilitating drug delivery. Mesoporous silica nanoparticles have previously been printed using soft-lithography and inkjet printing as a top-down method to create micro- and nanostructures [49]. However, deposition of the MSNs has not been studied for pharmaceutical applications. The purpose of this study was to develop a pharmaceutical MSN-based ink formulation, including a BCS class IV drug, to be deposited onto polyester transparency and hydroxypropylmethyl cellulose (HPMC) films using inkjet printing technology. The impact of drug-loaded compared to drug-free MSN–PEI was investigated with regard to (1) ink formulation development; (2) printability, as well as the (3) ink–substrate interactions. Attention was drawn to the print morphology regarding the drop and nanoparticle deposition patterns. Furthermore, a comparison of different drop deposition characterization techniques was also made. Here, we show that inkjet printing could be used for accurate deposition of pharmaceutical MSN ink suspensions according to predefined patterns, suggesting that the method could be utilized flexibly for screening purposes.

## 2. Results

### 2.1. Ink Development

**Figure 1:**
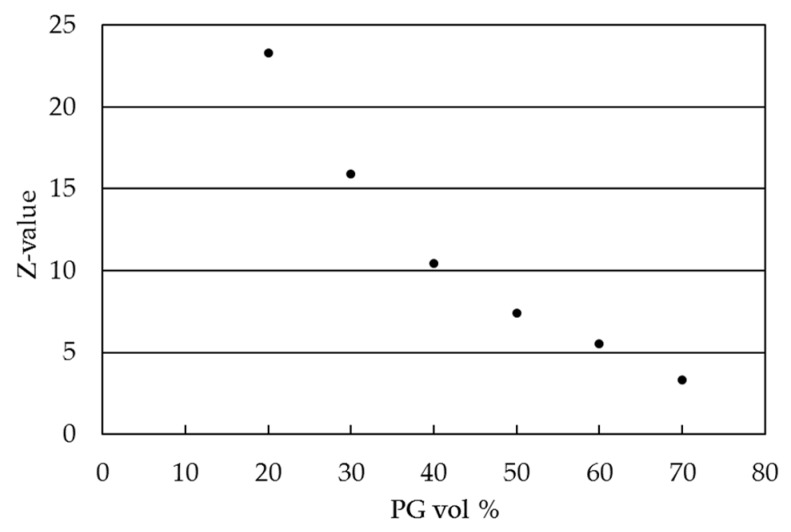
Ink formulation guidance: calculated Z-values of inks with 20–70 vol % of PG.

A pharmaceutical ink formulation consisting of MSNs as carrier vehicle for a BCS class IV model drug, furosemide (F), was developed. The polyethyleneimine surface-functionalized MSNs were loaded with 5 wt % (MSN–PEI–F5) and 15 wt % (MSN–PEI–F15) furosemide loading. MSNs are usually studied in aqueous buffer solutions resembling biologically relevant media. Consequently, an aqueous ink was chosen. Propylene glycol (PG) was added as an ink excipient to decrease the high surface tension of water, since a large amount of water might inhibit favorable droplet formation from inkjet printers [50]. PG was also added to increase the viscosity and move towards the ink viscosity values (8–20 mPas) recommended by the print head manufacturer [51]. The ink development was initiated by characterizing the physical fluid properties of the two chosen solvents in different ratios. An indication of printable combinations was gained by calculating the Z-value (Equation (1)), which relates the physical properties of the ink (density, ρ; surface tension, γ; viscosity, η), and the nozzle diameter (α) of the print head with the droplet formation (Figure 1, Supplementary Table S1) [50,52,53]. However, this value alone cannot be used to define jettability. This is because a variety of inks with different combinations of densities, viscosities, and surface tensions can have the same Z-value. A more accurate jettability prediction can be achieved if the velocity of the ejected droplet is taken into consideration when calculating the Z-value from the Reynolds (Re) and Weber (We) numbers (Equation (1)). However, in this study the velocity (*v*) of the ejected drop from the print head was not taken into consideration. Based on previous research, ink suspensions with Z-values between 5 and 9 have reported to be printable and have resulted in good drop deposition patterns [54]. Consequently, equal parts of distilled water (MQ) and PG (50/50) were chosen as the ink composition for further development.

(1) $$\begin{array}{c} Z=\frac{1}{Oh}=\frac{Re}{\sqrt{We}}=\;\frac{\sqrt{\alpha \rho \gamma }}{\eta }\\ Re=\frac{\nu \rho \alpha }{\eta }\\ We=\frac{\nu^{2}\rho \alpha }{\gamma } \end{array}$$

**Figure 2:**
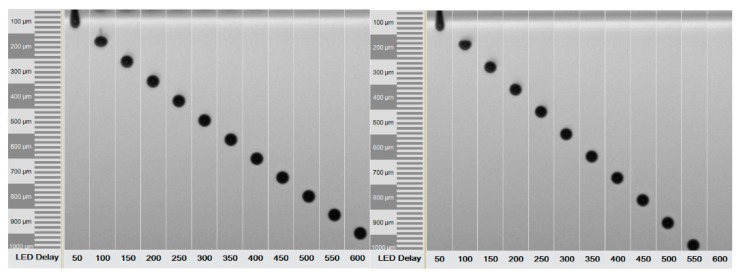
Droplet ejection and formation of the co-solvent mixture (MQ/PG) Z = 7.6 and 1 mg/mL nanosuspension (MSN–PEI–F15) Z = 7.8 monitored at 1600 Hz from the 50 µm Ø nozzle. The captures show the time (LED delay, µs, X-axis) vs. the travel distance (µm, Y-axis) of the recorded droplet ejection.

The physical fluid properties of the 1 and 5 mg/mL nanoparticle suspensions dispersed in MQ/PG were analyzed. The MSN suspensions showed Newtonian behavior at share rates from 10–1000 s^−1^. The average dynamic viscosity (*n* = 6) at 1000 s^−1^ (22 ± 0.5 °C) of both suspensions (1 & 5 mg/mL) was 6.2 mPas. Surface tension values of 43–45 mN/m (23 ± 0.5 °C) and densities of 1.043–1.044 g/cm^3^ were measured. Z-values of 7.6–7.8 were calculated for both the MQ/PG ink and the MQ/PG based drug-free and drug-loaded MSN suspensions. Due to this, a similar droplet ejection was recorded when identical print settings were applied (Figure 2).

### 2.2. Ink Characterization

The suspension consisted of spherical MSNs that were surface functionalized with the cationic polymer polyethyleneimine (PEI) to make the particles more easily dispersible in aqueous media and to protect the drug load from leaching out via so-called “molecular gate-keeping” [55]. A notable reduction in surface area and pore volume was characterized by nitrogen adsorption for the MSNs after the surface functionalization procedure (Table 1), suggesting a successful polymer accommodation onto the MSNs [56]. The pore diameter was also slightly reduced, which is in line with the expected polymer growth inside the pores. Thermogravimetric analysis (TGA) revealed that the PEI grafting amounted to 15 wt % (Supplementary Figure S1). Transmission electron microscopy (TEM) images of MSN and MSN–PEI were captured to confirm that the surface functionalization did not alter the fine structure of the particles (Supplementary Figure S2), which is not expected considering that the surface functionalization is conducted in an organic solvent.

Dynamic light scattering (DLS) measurements were performed to confirm the dispersibility of the unloaded and drug-loaded nanoparticles in the co-solvent mixture and to obtain the hydrodynamic particle size (Table 2). The analysis was only performed to ensure that the particle size of the suspensions were below the particle size recommendation (2 µm) set by the print head (Spectra SL) manufacturer [51]. We have previously noted that the hydrodynamic particle sizes obtained using DLS are considerably larger than the sizes measured by other techniques, especially for porous particles [57]. A low polydispersity index (PDI) value indicated a narrow particle size distribution and full dispersibility of the drug free MSN–PEI in the co-solvent mixture (Supplementary Figure S3). However, drug loading induced a higher tendency to formation of aggregates, denoted by the high PDI values. The high PDI values for the drug-loaded MSNs further suggest that the obtained hydrodynamic size values were not valid. However, the obtained size range proposes that the re-dispersibility was sufficient. A decrease in the net surface charge (ζ-potential) was also observed with increasing drug loading. Yet, the net positive ζ-potential values recorded for all suspensions indicated that the samples were electrostatically stabilized in the ink. Scanning electron microscopy (SEM) imaging of the empty (unloaded) MSN–PEI and drug-loaded MSN–PEI–F15 print deposits on transparency film was carried out to confirm the particle size from the prints (Supplementary Figure S4). The particle size of MSN–PEI and MSN–PEI–F15 were quantified as 306.6 ± 28.8 and 330.2 ± 57.3 nm, respectively.

**Figure 3:**
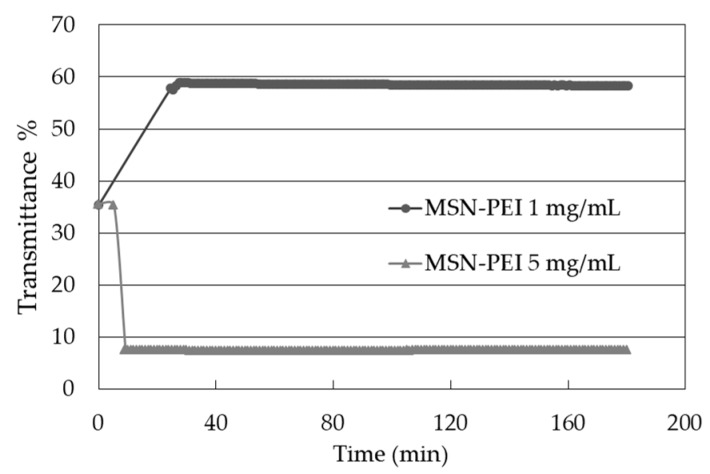
The mean transmittance (T%) of MSN–PEI 1 and 5 mg/mL measured with multiple light scattering over time.

**Figure 4:**
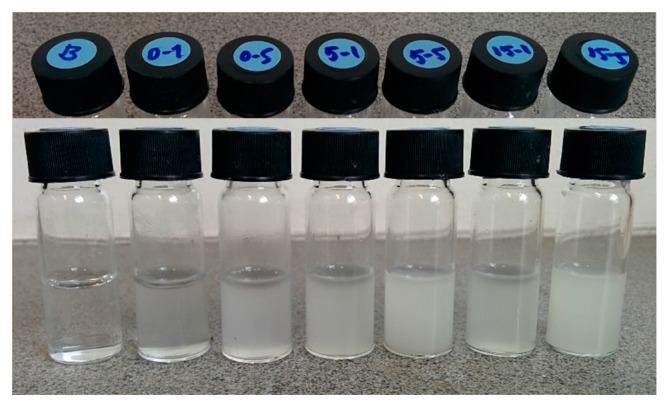
Images of MQ/PG (B) and of MSN–PEI (0–X), MSN–PEI–F5 (5–X) and MSN–PEI–F15 (15–X), X = 1 and 5 mg/mL.

The colloidal stability of the particle suspensions was characterized using the multiple light scattering (MLS) technique. The MLS results show an initial increase in the transmittance % (T%) for the 1 mg/mL MSN-PEI suspension, but a decrease for the 5 mg/mL MSN–PEI suspension (Figure 3). This behavior, starting from 35%, was also seen for the particle-free ink (MQ/PG, distilled water/polymer) before reaching equilibrium after mixing. The equilibrium was seen as a stable plateau and lasted for 180 min (total time of analysis). The drug loading as well as the particle concentration were seen to increase the turbidity of the samples (Figure 4).

### 2.3. Drug Loading and Drug Leaching into Ink

Measurements of the drug loading degree in the MSN–PEI systems were performed with a UV-Vis spectrophotometer using a five-point standard curve (λ_max_ = 273 nm, 2–25 µg/mL, R^2^ = 0.9998) in EtOH after drug elution from the drug-loaded MSNs. The drug loading was quantified as 0.39 wt % for F5 and 7.3 wt % for F15, respectively.

To make sure no premature drug leaching was taking place during printing, the drug release from the MSN–PEI into the ink solvent mixture was monitored (λ_max_ = 273 nm, 1–100 µg/mL, R^2^ = 0.9995) over 5 h for the 1 mg/mL F5 and F15 suspensions in MQ/PG. A stock solution was made consisting of furosemide in EtOH and dilutions were done using MQ/PG. No drug release was detected at the monitored wavelengths, indicating that the drug remained in the drug delivery vehicle during the processing steps.

### 2.4. Printing Process

**Figure 5:**
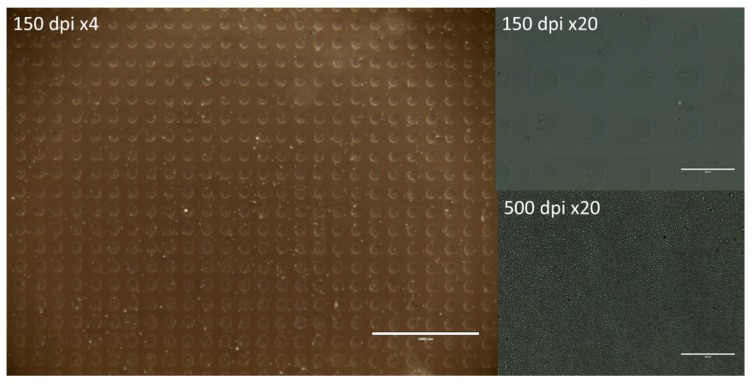
Light microscopy images of MSN–PEI 1 mg/mL suspensions printed with resolutions 150 dpi (×4 magnification, scale bar 1000 µm, ×20 magnification, scale bar 200 µm) and 500 dpi (×20 magnification, scale bar 200 µm) on HPMC films.

A piezoelectric DoD inkjet printer was used to print a digitally designed pattern of 1 × 1 inch^2^. The ink pressure was optimized and set at −19 mbar to avoid leaking and air suction into the ink container. A voltage of 85 V and waveform combinations of 4–5, 15–16, and 4–5 µs were applied to the piezoelectric element for uniform drop formation and ejection of the ink formulations (Figure 2). The substrate plate was heated to 30 °C to facilitate drying of the printed ink suspensions. Printing was performed on hydrophilic orodispersible HPMC films and hydrophobic polyester transparency films. Resolutions of 150 and 500 dpi were set (Figure 5). The lower resolution was used to generate samples for the ink–substrate interaction studies where separate and spatially distributed droplets could be distinguished. The higher resolution resulted in complete ink coverage of the predefined 1 × 1 inch print area. The higher resolution was used to prepare samples for dose quantification of the MSN–PEI–F15 prints on HPMC.

### 2.5. Dose Quantification

Nanoparticle drug delivery systems were printed with the MSN–PEI and MSN–PEI–F15 1 mg/mL suspensions onto HPMC films. An average furosemide dose of 6.7 ± 0.85 µg (λ_max_ = 273 nm) was quantified for the 10-layer MSN–PEI–F15 samples (*n* = 3) dissolved in EtOH, having the printed and drug-free 10-layer MSN–PEI sample as a blank for the UV-Vis spectroscopy measurements.

### 2.6. Characterization of Ink–Substrate Interactions

**Figure 6:**
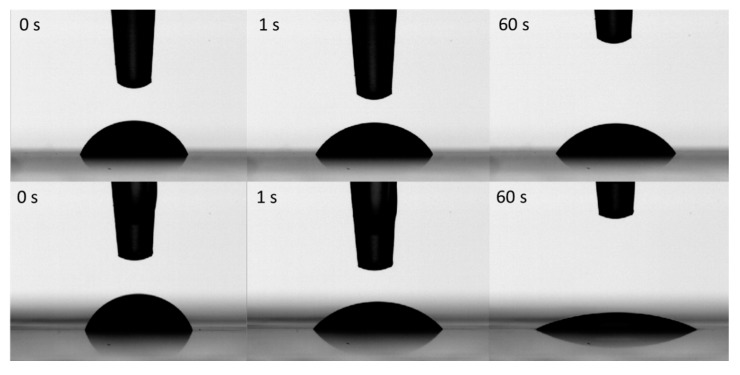
Contact angle of MSN–PEI (c = 5 mg/mL) on transparency (up) and HPMC (down) at 0, 1 and 60 s.

Hydrophilic HPMC and hydrophobic polyester transparency films were used as substrates. The surface roughness of the HPMC and transparency films, quantified by scanning white light interferometry (SWLI), was 0.06 ± 0.01 µm (*n* = 3) and 0.11 ± 0.02 µm (*n* = 3), respectively. Low contact angles (32°) were measured for all the aqueous ink formulations on the hydrophilic HPMC film compared to the higher contact angles (55°) on the hydrophobic transparency film, recorded after 60 s (Figure 6, Supplementary Figure S5).

**Figure 7:**
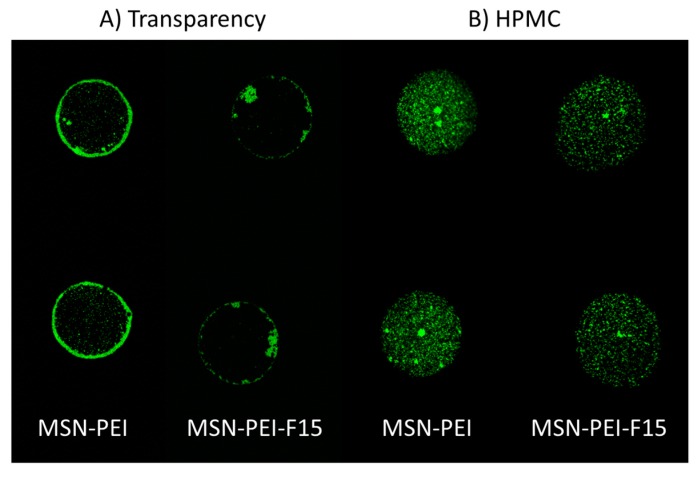
Confocal scanning laser microscope (CLSM) images of 5 mg/mL MSN–PEI and MSN–PEI–F15 suspensions printed with a resolution set at 150 dpi on (**A**) transparency and (**B**) HPMC films.

Confocal scanning laser microscopy (CLSM) was utilized to visualize the placement of the fluorescently labeled nanoparticles within the drop deposits on the transparency and HPMC films. The non-uniform nanoparticle distribution within the drop on the transparency film was a result of ink evaporation; the convective flow transported the particles towards the edges of the drop. This resulted in a pinning of particles at the contact line, leaving only a few particles in the center (Figure 7A). The drug-loaded particles seemed to have a greater adhesion towards each other compared to the unloaded MSN–PEI. Dissolution and gelling of the HPMC film surface as a means of suspension deposition resulted in entrapping of the deposited fluorescently labeled nanoparticles as the ink evaporated (Figure 7B). Based on the insufficient adherence of the particle to the transparency film and the drying behavior, additional experiments were only performed on HPMC.

**Figure 8:**
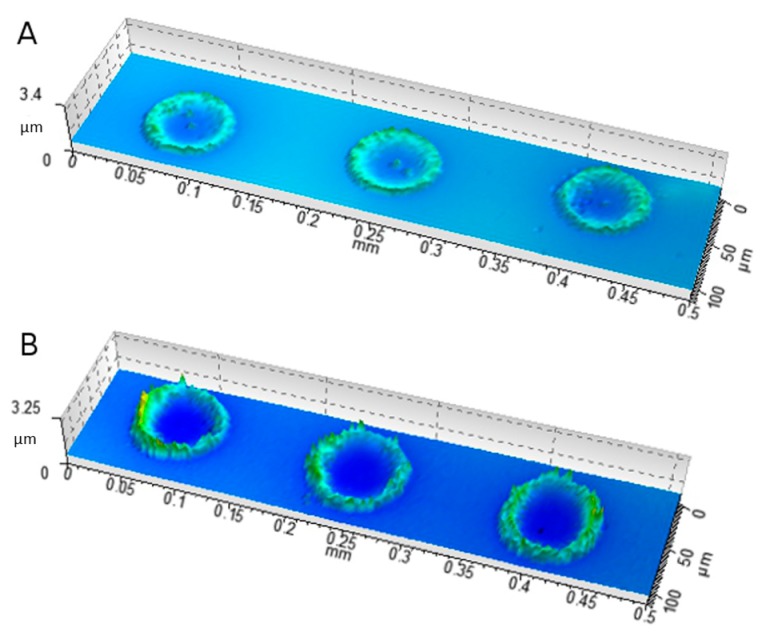
SWLI images of 150 dpi 5 mg/mL suspension deposits of (**A**) unloaded MSN–PEI and (**B**) drug-loaded MSN–PEI–F15 on HPMC.

SWLI measurements, with corresponding data analysis, were conducted to study the 3D profiles of the 150 dpi print deposits of the MQ/PG, the 1 mg/mL MSN–PEI and the MSN–PEI–F15 suspensions. Attention was drawn to the difference in height profiles of the ink (MQ/PG) and nanoparticle suspension (MSN–PEI) drop deposits (Table 3). Swelling of the HPMC film was observed as a means of ink (MQ/PG) deposition. The average step height of the nanoparticle suspension deposits was higher than the substrate swelling caused by the pure solvent. However, the step height was lower than the quantified size of the nanoparticles themselves. This was explained by the gelling behavior of the HPMC film surface reported above, which led to the incorporation of the nanoparticles on the film to some extent. A higher variability in step height was quantified for the drug-loaded MSN print deposits (data not shown). The deposits of the 5 mg/mL MSN–PEI and MSN–PEI–F15 suspensions are visualized in Figure 8. The 3D drop deposits were a result of both swelling of the film and particle aggregation. A more pronounced coffee ring effect of the drug-loaded particles on the HPMC film was distinguished from the 3D analysis. Smaller drop volumes, captured and analyzed using the advanced drop analysis program during the printing process, resulted in smaller drop deposit diameters gained from the SWLI analysis (Table 3).

**Figure 9:**
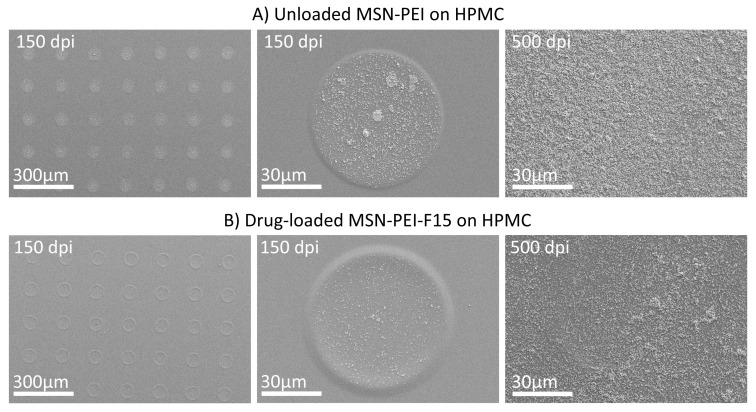
Ink deposits of (**A**) unloaded MSN–PEI and (**B**) drug-loaded MSN–PEI–F15 suspensions (5 mg/mL) with 150 dpi (one layer) and 500 dpi (five layers) with ×100 (300 µm) and ×1000 (30 µm) magnifications.

SEM analysis provided 2D information regarding the print result and the nanoparticle distribution within the droplet for the 150 dpi prints (Figure 9). Some agglomeration of the nanoparticles might have already occurred in the ink, but aggregation of the particles to a larger extent occurred upon drying of the single droplets. The higher print resolution (500 dpi) resulted in total ink coverage of the 1 × 1 inch print area. In general, SEM provided the same information as the CLSM. Yet, the method could be used for successful imaging of both non-labeled and fluorescently labeled nanoparticles.

## 3. Discussion

### 3.1. Ink Formulation

The composition of the ink formulation, in combination with the characteristics of the substrate (hydrophilic/hydrophobic), has a great impact on the (1) processing; (2) characteristics; and (3) applications of the inkjet-printed product. Furthermore, the choice and concentration of solvents, thickening agents, surfactants, solute, and particle size should also be considered [24,25,26,27,28,29].

PEI-functionalized MSNs was chosen as a drug delivery vehicle due to the previously reported good compatibility with cells (in vitro and in vivo) and due to the good dispersibility of the particles in aqueous media at physiological pH [48,58]. The aqueous media was also chosen as an ink solvent to avoid leakage of the poorly soluble drug from the loaded MSNs. PG, which is one of the most acceptable non-aqueous solvents for pharmaceuticals, was added to the ink formulation to ensure good processability [59]. This applies to previously reported general guidance on nanoparticle ink development for inkjet printing purposes [23]. Especially when developing inks for printing of electronics, the initial solvent should be chosen based on its capability to achieve high mass loading, while the second should be chosen to improve the jettability of the co-solvent system. The evaporation rate of the ink was and should in general be kept low to avoid nozzle clogging. Particle addition is known to increase the viscosity of the ink formulation and cause non-Newtonian flow properties of the ink formulation. Yet, the addition of up to 5 mg/mL was not seen to have any impact on the dynamic viscosity and the formulation was still showing Newtonian behavior, as did the particle-free ink. Similar observations has also been reported previously [26].

The properties of the nanoparticle formulation depend on the surrounding media. High positive ζ-potential values were measured for the nanoparticle formulations in the co-solvent mixture (MQ/PG), which is due to the positively charged PEI on the MSNs. The ζ-potential was seen to decrease with increased drug loading. Regardless of the decrease, all MSN suspensions (c = 0.2 mg/mL) were still considered electrostatically stable. The loading of the MSNs has also previously been reported to affect the net surface charge of nanoparticles in the suspension [60]. In general, weak acids (citric and lactic) have been suggested as good media for net positively charged nanoparticles to achieve stable nanosuspensions, and sugars (sucrose, dextrose, and mannitol) seem to be good media for net negatively charged nanoparticles. Isotonic salt solutions have been shown to be poor media from a suspension stability point of view. Since the hydrodynamic particle size and ζ-potential values were monitored at 0.2 mg/mL MSN concentration using DLS, (which was shown to not be a suitable method for the drug-loaded MSNs in the studied conditions), further characterization of the colloidal stability of the suspension was evaluated with MLS having samples with MSN concentrations corresponding to the printing formulations. Since the drawbacks of DLS are known, the obtained hydrodynamic particle size was only used to indicate if the particles could possibly be ejected from the 50-µm nozzles of the print head. The hydrodynamic size of nanoparticles depend not only on the size of the particle “core,” but also on any surface structure, as well as the type and concentration of any ions present in the medium. The high PDI values show that the particle sizes were not valid and thus the sizes of the drug-loaded particles should not be considered as actual sizes. Any change to the surface of a particle is known to affect the diffusion speed and change the apparent size of the particle. Consequently, interference of the drug with the MSN–PEI might have contributed to the poor data obtained for the drug loaded MSN–PEI–F5 and MSN–PEI–F15 samples. To achieve additional information about the particle size distribution and hydrodynamic size of the MSNs in the media, the angular dependence of the hydrodynamic radius gained by multiangular DLS and the radius of gyration obtained by static light scattering (SLS) could be studied in the future.

The colloidal stability of the 1 and 5 mg/mL MSN–PEI samples was confirmed by monitoring the suspensions using MLS for 180 min, which corresponded to the total printing time. The increase in T% for the 1 mg/mL MSN–PEI could be explained by the occurrence of repulsion between the particles, resulting in a clearer suspension after the first 30 min of standing before reaching equilibrium. The reason for the decrease in T% for the 5 mg/mL MSN-PEI suspension could be the high solid concentration resulting in flocculation.

Printing was successfully conducted with 1 mg/mL MSN, MSN–PEI, MSN–PEI–F5, and MSN–PEI–F15 suspensions. Dispersion and printing of particles without any surface functionalization were also possible. To print with the higher solid concentration was slightly more challenging and nozzle clogging was occasionally observed. The suspensions were prepared by dispersing the MSNs in MQ, followed by ultrasonication. Further sonication was done as PG was added to the formulation. Leaching of the drug into the ink was studied for 1–5 h, by centrifuging the MSN suspension prior to sampling and re-dispersing the particles by sonication. In the future, sonication times for new inks could be optimized to prevent drug leakage in the media if the suspensions need to be re-dispersed during processing. In this study no drug leakage could be observed and the particles were only dispersed once the ink suspension was prepared. However, after a longer storage, some sedimentation could be observed. Nevertheless, shaking or sonication of the suspension could easily re-disperse the systems.

Single solvent suspensions (especially aqueous) tend to result in coffee ring deposits, while co-solvent suspensions result in more homogenous print morphologies [26,29]. In this study, the coffee ring effect occurred on both substrates, regardless of having the non-volatile and drying rate-decreasing agent PG in the formulation. In order to avoid the coffee ring effect, the particle concertation could be decreased [26], another viscosity increasing/thickening agent could be added to inhibit the particles from travelling towards the contact line [29], or another substrate with other properties could be used. Furthermore, pre-treatment of the substrate as well as optimization of the drying conditions could influence the final morphologies of silica deposits [61]. A cross-linkable gel-forming agent could be added in the future if more extensive adhesion of the nanoparticles is needed or if the aim would be to print 3D drug delivery systems containing higher drug content.

### 3.2. Characterization Methods

**Figure 10:**
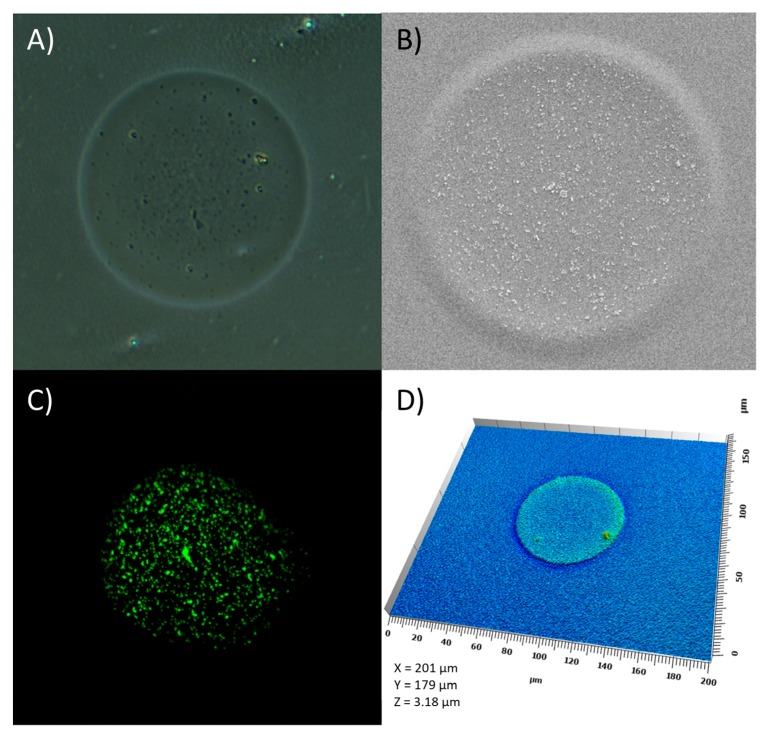
Imaging methods of droplets: (**A**) Light microscope, 1 mg/mL MSN–PEI–F15; (**B**) SEM, 5 mg/mL, MSN–PEI–F15; (**C**) CSLM, 5 mg/mL MSN–PEI–F15, (**D**) SWLI, 1 mg/m MSN–PEI–F15.

Characterization of the print morphologies is of interest especially when the aim is to manufacture nano- and microstructures according to a top-down approach. Both destructive (CSLM, SEM) and/or non-destructive (light microscopy, SWLI) characterization methods can be utilized (Table 4). The light microscope was a fast method and gave an initial idea of the print result (Figure 10A). Since the wetting capacity of an ink depends on the properties of the substrate (i.e., surface energy), the instrument could be used to study at what resolution the drops merge and form a uniform ink layer on a specific substrate. The nanoparticle distribution of the area totally covered by ink as well as the spatial drop deposition pattern and nanoparticle distribution within the drops were successfully imaged by SEM (Figure 10B). The particle size of the top layer consisting of deposited MSN–PEI and MSN–PEI–F15 on transparency film was easily quantified from the SEM images (Supplementary Figure S4) using the ImageJ software. Yet this was not optimal for analysis of the nanoparticles printed onto HPMC film as it started melting when the magnification was increased. The distribution of the fluorescently labeled nanoparticles of the prints was successfully imaged using CSLM (Figure 10C). However, one has to bear in mind that only one focal layer is imaged at a given height. Consequently, the coffee ring effect that was present on both transparency and HPMC was only seen on one substrate. The emitted fluorescence of the drug-loaded MSN–PEI–F15 prints was observed to be weaker than the fluorescence of the unloaded MSN–PEI deposits. This phenomenon has previously been reported for similar MSN–PEI particles, explaining the occurrence by a slight decrease in pH [48]. This observation reveals that the pH of the ink changed due to the drug loading of the particles. SWLI provided non-destructive characterization of the 3D structures of the print morphologies (Figure 10D). However, SWLI did not reveal any MSN particle uniformity information due to the nature of optical transparency of the silica material.

### 3.3. Current Trends in MSN–Cell Interaction Studies and Potential Screening Platforms

Over the past decade, an increasing number of publications regarding nanosafety have been published. Despite this, the transition from pre-clinical to clinical trials is still slow for the nanopharmaceuticals since there is no clear consensus regarding (1) definitions or classification in nanotechnology or (2) characterization techniques to ensure safe nanomedicines for human [31,62]. In particular, an understanding of the interactions at the nano–bio interface (by evaluation of both the synthetic and biological identity of the complex formulations) is needed before clinical trials can be safely conducted [63].

The possibility of accurate and well-controlled deposition of pharmaceutical MSN–PEI formulations using inkjet printing technology, as shown in this study, forms the basis for further exploration of interactions at the nano–bio interface. Investigations regarding cell exposure to MSN-covered substrates, implants, and drug delivery systems prepared using different techniques, such as dip coating [64,65] and spin coating [66], have been performed previously. To study the impact of nanopharmaceuticals and biomaterials on cells has proven to be important since the morphological properties of the surface (i.e., surface roughness) are seen to influence the cell morphology and metabolism [67,68]. Inkjet printing is a non-contact, accurate, and more flexible method than dip coating. Nanoparticle coverings can easily be formed by spin coating, but the method does not allow for the deposition of the nanoparticle formulation according to predefined patterns. In short, inkjet printing technology and the ink formulation presented in this study could be used if a non-contact method, specific print designs, patterning, spatial control, or manufacturing flexibility is wanted.

Development of drug delivery systems using digital printing technologies goes well with the current trend of personalized medicine in the medical and pharmaceutical fields due to the abovementioned advantages and the possibility of on-demand and point-of-care manufacturing [69]. Research efforts towards personalized medicine are also evident in the development of nanomaterials for in situ monitoring of drug delivery, drug release, and drug efficacy [70]. Nanomaterials have also been mentioned in the field of super generics, where development of personalized and cost-efficient products is a focus in the drive to meet the needs of the healthcare sector and patients [32].

Printing has previously been adapted not only for manufacturing of drug delivery systems; different digital printing technologies such as inkjet, microextrusion, and laser-assisted printing have also been used for cell, tissue, and organ printing [71]. The physical characteristics of the material to be printed usually dictate the method of choice [72]. Hepatocytes have, for instance, been printed to create “liver-on-chip” models [73]. Printing of epithelial cells has been conducted and the method has also been suggested as applicable for biological and biomedical studies to gain more understanding about e.g., cell–cell communication and stimulus response [74].

## 4. Materials and Methods

### 4.1. Synthesis of MSNs

The MSNs were synthesized in an aqueous basic solution with the addition of absolute methanol (HPLC gradient grade, J.T. Baker, Philipsburg, NJ, USA) as co-solvent. Cetyltrimethylammonium chloride (CTAC solution, 25 wt % in H_2_O, Sigma-Aldrich, Steinheim, Germany) was dissolved in the basic reaction solution and served as a structure-directing agent (SDA). Tetramethylorthosilicate (TMOS, 99%, Sigma-Aldrich) was added to the reaction solution as silica source. The reaction was conducted in a conical flask overnight under stirring at room temperature. The molar composition in the synthesis solution was TMOS:CTAC:NaOH:MeOH:H_2_O (1:1.4:0.3:1433.7:3188.7). Sodium hydroxide (NaOH) was purchased from Merck KGaA, Damstadt, Germany. Fluorophore labeling of MSNs was carried out according to the protocol of our previous work [48]. Briefly, a mixture of fluorescein isothiocyanate (FITC, Sigma-Aldrich) and aminopropyl trimethoxysilane (APTMS, Sigma-Aldrich) (FITC:APTMS, 1:3) was pre-reacted in methanol for 30 min and added to the synthesis solution. Finally, the silica source TMOS was added (TMOS:APTMS, 100:1). The formation of the particles took place overnight under continuous stirring at room temperature.

After the particle synthesis was accomplished, the structure-directing agent was removed by washing the particles with an extraction solvent. The extraction solvent was a 1:8 mixture of HCl (37–38%, J.T. Baker, Philipsburg, NJ, USA) and ethanol (99.5%, Altia Oy, Helsinki, Finland). The dispersion was centrifuged and the supernatant was removed and replaced with fresh extraction solvent. The solution system was sonicated for 30 min after which the washing cycle, starting with centrifuging, was repeated three times. After the three cycles d, pure ethanol was added to wash away the extraction solvent. Half of the particles were kept for drying in vacuum whereas the other half was stored as an ethanol dispersion for further polyethyeleneimine (PEI, Sigma-Aldrich) surface functionalization. The surface of the MSNs was modified with PEI by the surface growing method according to our previously described protocols and the samples were named as MSN-PEI in the study [56,75,76]. The prepared samples were kept in the fridge as an ethanol suspension. MSN-PEI particles with and without FITC label was used for drug loading in the study.

### 4.2. Characterization of MSN and MSN-PEI

The dispersibility of the unloaded and drug-loaded MSN-PEI in the solvent mixture, the hydrodynamic particle size (Z-average, intensity) and ζ-potential were analyzed with a DLS/ζ-potential instrument (Malvern ZetaSizer NanoZS, Malvern Instruments Ltd., Malvern, UK). The efficiency of the functionalization was evaluated based on the ζ-potential data. The weight percentage of accommodated PEI in respect to MSN was determined using thermogravimetric analysis (TGA, STA 449 F1 Jupiter, NETZSCH, Selb, Germany). The analysis was performed over a temperature range of 25 °C to 770 °C. Nitrogen adsorption (Autosorb-1, Quantachrome Instruments, Boynton Beach, FL, USA) was performed to evaluate the surface area, pore size and pore volume or in general the porosity of the particle. The fine structure of MSN and MSN-PEI particles were analyzed by transmission electron microscopy (JEOL JEM-1400 Plus, JEOL Ltd., Tokyo, Japan). The particle size of the printed MSN-PEI and MSN-PEI-F15 (*n* = 30) on transparency was quantified using the SEM pictures and the image analysis program ImageJ (v. 1.50i 2011, National Institutes of Health, Bethesda, MD, USA).

### 4.3. Drug Loading

The BCS class IV drug, furosemide, F (Ph.Eur., Fagron Nordic, Copenhagen, Denmark) was chosen as a model drug to be loaded into the MSN-PEI samples in this study. The drug loading was carried out using the solvent immersion method. In this process 5 wt % and 15 wt % of F with respect to the MSN-PEI mass was soaked into a cyclohexane solution that contained MSN-PEI to obtain particles with 5 and 15 wt % loading degrees (abbreviated as MSN-PEI-F5 and MSN-PEI-F15, respectively). The drug-loaded suspensions were ultrasonicated and kept in a rotating wheel mixer overnight at room temperature. After adsorption of F, the F loaded MSN-PEI samples were centrifuged and the obtained precipitates were vacuum-dried. Afterwards, F elution was carried out in order to determine the amount of drug-loaded on MSN-PEI. The loading degrees were investigated by preparation of 1 mg/mL suspensions in EtOH (Etax Aa 99.5%, Altia Oy). The suspensions were held for 30 min in a sonication bath and an additional 90 min in a rotating wheel mixer (50 rpm) protected from light. The suspensions were centrifuged (8000 rpm, 10 min) and the drug content was measured from the supernatant after dilution in EtOH.

### 4.4. Ink Preparation and Characterization

The 1 and 5 mg/mL nanoparticle suspensions were prepared by dispersing the MSN and MSN-PEI reverse-osmosis purified water (distilled water, MQ) using a Covaris Acoustic Ultrasonicator (Covaris, Brighton, UK). Propylene glycol (PG, ≥99.5%, Sigma-Aldrich), was added as a humectant and stabilizing agent to the suspension during sonication, after which proper dispersion of the particles in the aqueous phase was obtained. The solvent mixture consisted of equal volumes of MQ and PG. The solvent mixture MQ/PG and the MSN-PEI 1 and 5 mg/mL nanosuspensions were characterized with regard to their physical fluid properties as described below.

#### 4.4.1. Dynamic Viscosity

The dynamic viscosity was measured using a stress controlled rheometer (Physica MCR 300, Anton Paar, Graz, Austria, Software: Rheoplus), connected with a refrigerator bath and a temperature control unit (Techne RB-12A & TU-16D, Vernon Hills, IL, USA) and equipped with a double gap measurement geometry (DG26.7/T200/SS, internal ø: 24.655 mm, external ø: 26.669 mm, concentricity: ±8 µm). The dynamic viscosity of the inks was monitored after sample conditioning @ 22 ± 0.5 °C by application of a shear stress ramp at rates of 10, 100, and 1000 s^−1^.

#### 4.4.2. Surface Tension and Density

A contact angle goniometer CAM 200 (KSV Instruments Ltd., Espoo, Finland, later Biolin Scientific) was used to measure the surface tension of the inks at room temperature (23 ± 0.5 °C). The pendant drop method was applied to measure the surface tension of the inks. A 5-µL drop was dispensed from a disposable plastic tip (Fintip 200 µL, Thermo Scientific, Vantaa, Finland) and imaged for 10 s. The recorded drop shape was fitted to the Young–Laplace equation using the OneAttension software (Theta1.4) to calculate the surface tension of the ink. The density of the suspensions was measured by weighing 1 mL of 23 ± 0.5 °C suspensions and calculating the density according to the obtained weight.

#### 4.4.3. Colloidal Stability of MSN-PEI Suspensions

The 1 and 5 mg/mL MSN-PEI suspensions were irradiated in the near infrared region (air = 850 nm) with an electroluminescent diode using multiple light scattering (MLS, Turbiscan MA2000, FormulAction, Tolouse, France) to evaluate the colloidal suspension stability. Sampling was performed every minute during a total sampling time of 180 min from the 7-mL sample (25 °C, *n* = 1). The results were analyzed using the Turbisoft software (v 1.2.1, FormulAction, CIRTEM, Tolouse, France), generating mean transmission profiles.

#### 4.4.4. Drug Release in Ink

The drug release from the MSN-PEI-F5 and F15 particles (c = 1 mg/mL, *n* = 3) into the ink was studied for 5 h. The particles were dispersed in MQ/PG and mixed in a rotating wheel mixer (50 rpm) protected from light. An aliquot of 2 µL was withdrawn every hour from the ink supernatant, gained by centrifuging the samples at 5000 rpm for 5 min. The ink suspensions were re-dispersed by vortexing and sonicating them after each sample withdrawal. Drug quantification was performed in the pendant mode of a UV-Vis spectrophotometer (Nanodrop 2000c spectrophotometer, Thermo Scientific) at λ_max_ 273 nm.

### 4.5. Inkjet Printing

A piezoelectric inkjet printer, (PixDro LP50, Meyer Burger, Eindhoven, The Netherlands) equipped with a Spectra SL 128 AA, print head (nozzle Ø 50 µm, Fujifilm Dimatix Inc, Santa Clara, CA, USA), was used for sample preparation. The suspension was introduced to the ink container with a syringe without any filtration. Monitoring of working nozzles and droplet ejection was performed using a high-speed time-of-flight camera that was attached to the printer. Droplet formation was studied using the advanced drop analysis software (ADA, v.2.3, PixDro) by monitoring the droplet at 50–600 µs after ejection at a frequency of 1600 Hz. The print settings (ink pressure, voltage, and waveform) were optimized. Printing was performed at a speed of 200 mm/s, using one nozzle in the print head with the resolution set at 150 and 500 dpi. The droplets selected for the printing task were checked and analyzed with regard to their volume (*n* = 10) before every printed layer with the help of a high-speed camera. The “print view” was calibrated according to a three-image calibration procedure given by the manufacturer of the printer. Drop volume calculations were performed using the PixDro software based on a snapshot of the ink droplet. Substrate plate heating was set at 30 °C to facilitate drying of the deposits during the printing. The samples were stored at room temperature and protected from light. The printed doses (500 dpi, 10 layers) were left to dry at room temperature.

### 4.6. Substrate

The inks were printed onto orodispersible hydroxypropylmethyl cellulose (HPMC) and polyester transparency films (Folex Imaging, Clear transparent X-10.0 films). The orodispersible films consisted of HPMC (15 wt %, Pharmacoat 606, Shin Etsu, Tokyo, Japan), glycerol (3 wt %, Sigma-Aldrich), and purified water and were cast using a film applicator (Multicator 411, Erichsen GmbH & Co. KG, Hemer, Germany), with the wet thickness set at 500 µm.

### 4.7. Quantification of the Prints

The BCS class IV model drug furosemide incorporated in the MSN-PEI was quantified from the MSN-PEI-F15 10 layer prints using a UV-Vis spectrophotometer (Nanodrop 2000c spectrophotometer, Thermo Scientific, Wilmington, MA, USA) at λ_max_ 273 nm. The 1 × 1 inch samples (*n* = 3) were cut into four parts and placed in 1 mL of EtOH (99.9%, Etax Aa, Altia, Helsinki, Finland) in an Eppendorf vial. The samples were sonicated in a 25 °C water bath for 30 min and kept in a rotating wheel mixer for an additional 1.5 h. The MSNs and the HPMC film were centrifuged for 10 min in EtOH at 8000 rpm. The drug-free MSN-PEI prints were treated in the same manner and served as blank for the UV-Vis measurements.

### 4.8. Contact Angle

Contact angle measurements of the solvent mixture and the 1 and 5 mg/mL nanosuspensions (23 ± 0.5 °C) were performed on the transparency and HPMC films according to the sessile drop method by applying a 5-µL drop of ink onto the films in triplicate and monitoring the contact angle for 60 s. The measurements were performed using the same instrument as for the surface tension measurements described in Section 4.4.2.

### 4.9. Visual Characterization of the Prints

The 1 and 5 mg/mL MSN-PEI and MSN-PEI-F deposits were characterized by confocal laser scanning microscopy (CLSM), scanning electron microscopy (SEM), optical microscopy, and scanning white light interferometry (SWLI).

#### 4.9.1. Confocal Laser Scanning Microscopy

The prints were imaged using a Leica TCS SP 5 confocal scanning laser microscope (CLSM, Leica Microsystems GmbH, Wetzlar, Germany, Lenses: HCX PL APO 40×/1.15 and 63×/1.32 oil objectives) with an excitation wavelength of 488 nm.

#### 4.9.2. Scanning Electron Microscopy

Scanning electron microscopy (SEM, LEO Gemini 1530, Carl Zeiss Microscopy GmbH, Oberkochen, Germany) was used to image the prints. The samples were pretreated with a carbon layer. Images were recorded at an acceleration voltage of 5 kV using the secondary electron detector. Images of 50, 100, 250, and 1000 time magnifications of the drop deposits, corresponding to a Polaroid 545 print with the image size of 8.9 × 11.4 cm, were captured.

#### 4.9.3. Optical Microscopy

An optical microscopy imaging system (Evos XL Core Imaging System, Fisher Scientific GmbH, Schwerte, Germany) was used to image the prints using ×4, ×20, and ×40 magnification lenses (LPlan PH2).

#### 4.9.4. Scanning White Light Interferometry

Scanning white light interferometry (SWLI) was used to obtain 3D images of the prints. All samples were imaged using a custom-made scanning white light interferometer instrument. The instrument featured a NIKON reflective microscope frame, equipped with a NIKON IC Epi Plan DI 10× MIRAU interferometry objective (Edmund Optics Ltd., Nether Poppleton, York, UK), a 100 μm piezoelectric z-scanner (Physik Instrumente P-721 PIFOC^®^, Karlsruhe, Germany), a high-resolution CCD camera (Hamamatsu Orka Flash 2.8 CMOS, Hamamatsu City, Japan), and two motorized translation stages (STANDA 8MTF-102LS05, Vilnius, Lithuania). As a white light source, a standard halogen lamp was used. The total magnification of this setup was ×6.3.

Scanning and data acquisition was controlled with a C++ based software built in-house. 3D image construction and 3D data analysis (e.g., determination of deposition diameter and deposition thickness) were performed using the commercial MountainsMap^®^ Imaging Topography 7.4 software (Digital Surf, Besançon, France).

Three different 1 × 1 inch areas of each of the printed samples of interest were imaged with the SWLI instrument without any further sample pretreatment. If necessary, the samples were flattened using small weights to minimize the waviness originating from the film substrates. The 1 × 1 inch printed samples were imaged both in the center parts and in the peripheral parts of the printed area.

## 5. Conclusions

This proof-of-concept study showed that it is possible to formulate and print pharmaceutical nano-ink suspensions containing unloaded and drug-loaded MSNs using digital, non-contact, inkjet printing technology. The ink suspensions remained physically stable during the processing steps and printing time. No premature drug leakage from the nanoparticles was observed. This study has proven that the ink formulation and the substrate properties together affect the final MSN distribution and morphology of the ink deposits. Different 2D and 3D characterization methods of the MSN prints were also evaluated. Versatility in the design of a screening platform for drug delivery systems can be achieved on a (1) nanoparticulate and (2) 3D design level; the design possibilities of the MSNs and inkjet printing are extensive. The presented technology and concept could be utilized in detailed investigations of the nano–bio interface in the future when used as a cell substrate. When put in a larger scope, drug delivery systems could be printed on demand and at the point of care to meet the emerging needs of healthcare systems, policymakers’, and patients’ by introduction of a more personalized treatment and development of more cost-efficient products in the form of e.g., super generics.

## Figures and Tables

**Table 1:** Surface area, pore volume and pore size of the MSN and MSN–PEI particles.

Particle	Surface Area (m^2^;/g)	Pore Volume (cm^3^;/g)	Pore Diameter (nm)
MSN	1882	1.87	4.09
MSN–PEI	930	0.82	3.54

**Table 2:** Hydrodynamic particle size, polydispersity index (PDI), and ζ-potential of the drug-free and drug-loaded MSN–PEI suspensions (c = 0.2 mg/mL) in MQ/PG. The data is presented in triplicate ± StD.

Sample	Particle Size (nm)	PDI	Zeta Potential (mV)
MSN–PEI	395.4 ± 1.6	0.050 ± 0.026	57.0 ± 0.6
MSN–PEI–F5	289.0 ± 13.5	0.802 ± 1.07	46.6 ± 0.6
MSN–PEI–F15	445.9 ± 117.1	1.00	38.5 ± 2.8

**Table 3:** Average drop volume, diameter and step height of the MQ/PG ink and 1 mg/mL MSN–PEI suspension deposits on HPMC.

	MQ/PG	MSN–PEI (1 mg/mL)
Average step height (nm) *n* = 9	60.5 ± 24.1	155.0 ± 17.8
Average drop volume (pl) *n* = 3	52.9 ± 0.6	54.9 ± 0.2
Average drop diameter (µm) *n* = 12	81.2 ± 2.2	85.8 ± 1.6

**Table 4:** Characterization methods for drop deposits.

	Light Microscope	SEM	CSLM	SWLI
	2D	2D	2D	3D
Non-destructive	+	−	−	+
Droplet deposition	+	+	+	+
Drop diameter	+/−	+	+	+
MSN uniformity	−	+	+	−

## Supplementary Materials

The supplementary materials are available online.
