# Supplementary material for: Inkjet Printing of Drug-Loaded Mesoporous Silica Nanoparticles—A Platform for Drug Development
Source: Molecules. 2017 Nov 21;22(11):2020. doi: 10.3390/molecules22112020 (PMC6150386; doi:10.3390/molecules22112020)
Supplement: Supplementary file 1 [file molecules-22-02020-s001.pdf]

Supplementary Table S1. Ink properties.

	Dynamic viscosity (mPas) @ 1000 s^{-1}, 22 ± 0.5 °C	Surface tension (mN/m) @ 23 ± 0.5 °C, 1 px = 1.0576 × 10^{-5}	Density (g/cm^3) @ 23 ± 0.5 °C
Ink (blank)	6.27	43.73 ± 0.34<br>44.82 ± 016 *	1.043
MSN 1 mg/ml	6.15	44.55 ± 0.52	1.044
MSN 5 mg/ml	6.21	43.59 ± 0.52	1.044
MSN–PEI 1 mg/ml	x	45.24 ± 1.16 *	x
MSN–PEI 5 mg/ml	x	45.34 ± 0.41 *	x
MSN–PEI–F5 1 mg/ml	x	44.91 ± 0.51	1.043
MSN–PEI –F15 1 mg/ml	x	44.55 ± 0.69	1.044
MSN–PEI–F5 5 mg/ml	x	44.42 ± 0.56	1.044
MSN–PEI –F15 5 mg/ml	x	43.05 ± 0.16	1.043

* 1 px = 1.0797 × 10^{-5}

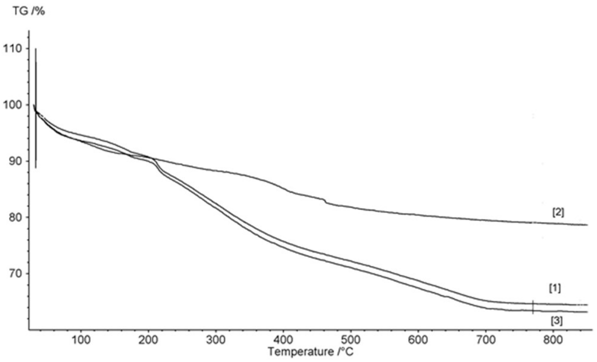

Supplementary Figure S1. TGA results of PEI surface functionalized MSNs in toluene [1] and [3] and base MSNs [2].

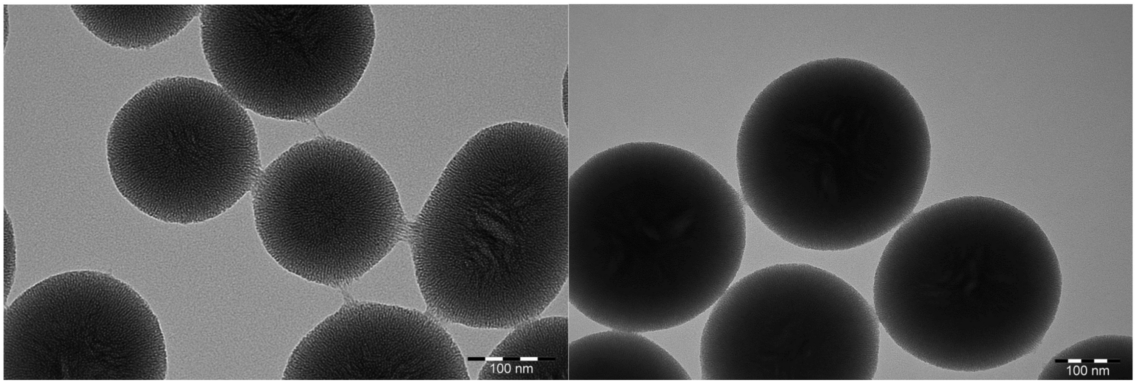

a b

Supplementary Figure S2. TEM images of **a**) MSN without surface functionalization and **b**) MSN-PEI, scale bar 100 nm.

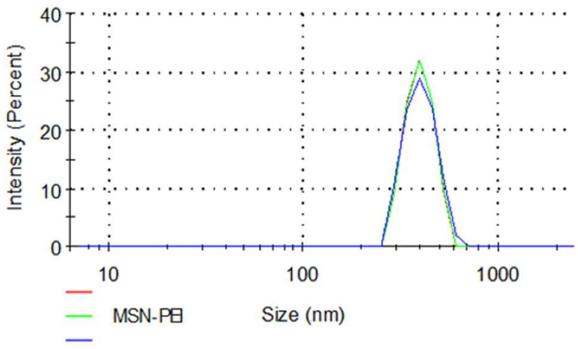

Supplementary Figure S3. The hydrodynamic particle size of MSN-PEI dispersed in MQ/PG (n=3) measured using DLS.

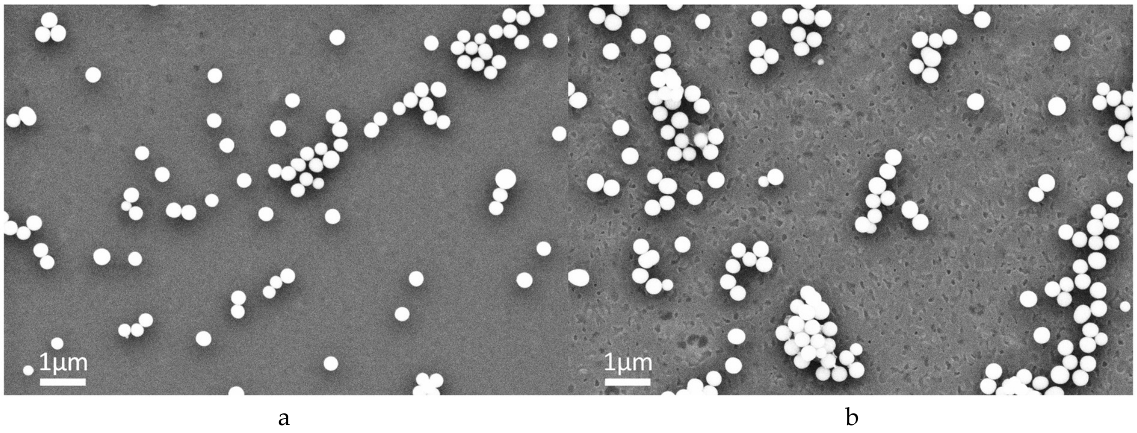

Supplementary Figure S4. SEM images of **a**) unloaded MSN-PEI *on the left* and **b**) drug-loaded MSN-PEI-F15 *on the right* printed on transparency film (scale bar 1 µm).

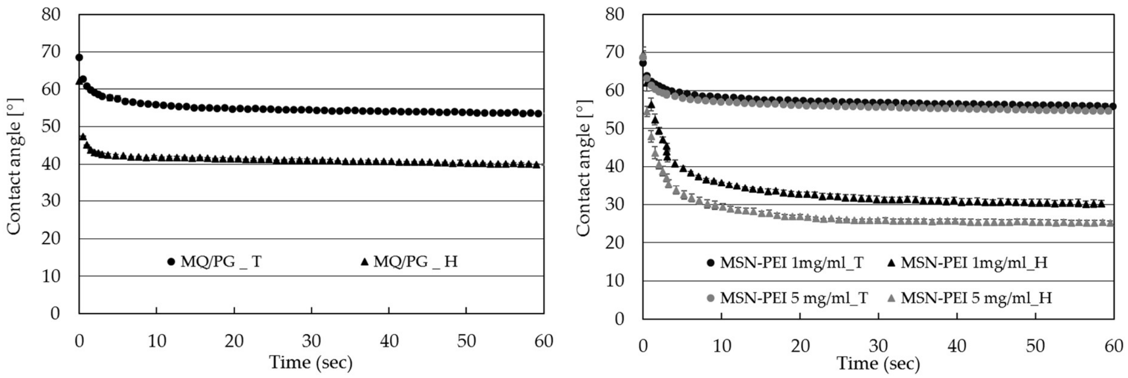

Supplementary Figure S5. Contact angle [°] versus time (sec) of MQ/PG, 1 mg/ml MSN-PEI and 5 mg/ml MSN-PEI, (n = 3) on Transparency (T) and HPMC (H) films.
